# A Study on Drug-Drug Interaction of Esomeprazole and Anti-Diabetic Drugs

**DOI:** 10.4103/0975-1483.71624

**Published:** 2010

**Authors:** VKM Swamy, RS Setty, MM Shankaraiah, TM Jyothi, SV Rajendra

**Affiliations:** *Department of Pharmacology, S.C.S. College of Pharmacy, Harapanahalli - 583 131, Karnataka, India*; 1 *College of Clinical Pharmacy, King Faisal University, Alahsa - 31982, Kingdom of Saudi Arabia*

**Keywords:** Drug–drug interaction, esomeprazole, glibenclamide, tolbutamide

## Abstract

Drug–drug interaction between esomeprazole at therapeutic and higher doses and sulfonylureas was studied. Sulfonylureas (tolbutamide 40 mg/kg and glibenclamide 40 µg/kg) were administered and the time to onset of hypoglycemia, the duration of the hypoglycemia, and the peak hypoglycemia were determined. Esomeprazole (1.8 mg/kg, 3.6 mg/kg, and 30 mg/kg) was administered for 8 days and its influence on sulfonylurea-induced hypoglycemia was studied. Therapeutic doses of esomeprazole, i.e., 1.8 mg/kg and 3.6 mg/kg dose did not influence the hypoglycemia induced by sulfonylureas. However, a higher dose, i.e., 30 mg/kg, did significantly enhance the duration of hypoglycemia and the peak hypolgycemia. Esomeprazole (30 mg/kg) by itself did not reduce the blood glucose levels; therefore, a pharmacodynamic type of drug interaction can be ruled out. Similarly, a pharmacokinetic type of drug interaction may be ruled out at therapeutic doses. The CYP isoenzyme system involved in the metabolism of sulfonylureas are not very sensitive to esomeprazole and the dose and frequency of administration of sulfonylurea need not be readjusted when they are used concomitantly with esomeprazole (at therapeutic doses).

## INTRODUCTION

Drug–drug interactions may occur when more than one drug is administered in a patient to treat a single ailment or multiple ailments. These concomitantly used drugs may either cause pharmacodynamic or pharmacokinetic types of interactions. The net result of both the types of interactions is the alteration in the therapeutic effect of either or both the drugs. There are several diseases that require treatment for the lifetime, e.g., diabetes and hypertension. Patients with such diseases will often need to be administered drugs for treatment of other co-existing diseases, either for a short period or lifelong. There is then a possibility of occurrence of interactions between drugs, resulting in either reduced or enhanced effects of any of the drugs. Therefore, monitoring and readjustment of the dose/s is often necessary to optimize treatment. In the present study, two diseases (diabetes and gastric ulcers) that may co-exist and require chronic treatment were considered and the occurrence of interaction between the concurrently used drugs was assessed

Diabetes mellitus is a disease characterized by elevated blood glucose levels. It requires treatment for prolonged periods, usually lifelong. Diabetic patients may also have other diseases, e.g., peptic ulcers, infectious diseases, etc. In such situations, treatment for the different ailments will have to be given simultaneously. Peptic ulcer is one such disorder that requires treatment for a prolonged period. There are several patients who suffer from both diabetes and peptic ulcers. In such patients, H_2_-receptor blockers or proton pump inhibitors are administered concomitantly with sulfonylureas or insulin preparations. There are reports that H_2_-receptor blockers such as ranitidine inhibit the metabolism of sulfonylureas and enhance their bioavailability.[1,2] Similarly, there are reports that chronic usage of omeprazole increases the peak concentration and apparent elimination half-life of phenytoin in healthy male volunteers.[3] CZP2C9 is the enzyme responsible for the metabolism of phenytoin and sulfonylureas. Therefore it is hypothesized that esomeprazole may influence the metabolism of study drugs. In addition, there is a report that omeprazole increased the duration of hypoglycemia and peak hypoglycemia induced by sulfonylureas in healthy albino rabbits.[4] One report has been published showing that proton pump inhibitors like lansoprazole induce the cytochrome-P450 enzyme system.[5] Lansoprazole at two dose levels – 30 mg/kg and 60 mg/kg for 7 days – significantly enhanced the duration of hypoglycemia induced by tolbutamide and the peak hypoglycemia slightly; the time to onset of hypoglycemia was not altered significantly. Similarly, pretreatment with lansoprazole has been shown to enhance the peak hypoglycemia and the duration of hypoglycemia induced by glibenclamide in healthy rabbits.[6] Pretreatment with pantoprazole, 10 mg/kg for 7 days, increased the peak hypoglycemia and the duration of action of glipizide in healthy rabbits and rats as well as in diabetic rats.[7] However, there are no such reports regarding interactions between esomeprazole and antidiabetic agents. Hence, the present study planned to assess the interaction between esomeprazole and oral antidiabetic agents such as tolbutamide and glibenclamide.

## MATERIALS AND METHODS

The esomeprazole used in this study was from Micro Labs Ltd., Bangalore; the tolbutamide from Albert David Ltd., Bombay; and the glibenclamide from Aventis Pharma Ltd., Goa.

### Animals

Albino rats of either sex, weighing 150–250 g, were procured from Sri. Venkateshwara Enterprises, Bangalore, and were stored under standard husbandry conditions. They were used for the study after a 7-day acclimatisation period. Permission for the usage of animals and approval of the experimental protocols for the study was obtained from the institutional animal ethics committee prior to the experimentation. The Registration No. for institutional animal house is 157/1999/CPCSEA.

### Experimental procedure

Albino rats of either sex (150–250 g), maintained under standard conditions, were randomly distributed into 6 groups of 6 animals each. The experiment was conducted in two phases. In the first phase, after fasting for 18 hours all the animals of groups 1, 2, and 3 were administered tolbutamide (40 mg/kg) and groups 4, 5, and 6 were given glibenclamide (40 µg/kg) orally. Zero-hour blood samples were collected for estimation of fasting blood glucose levels. Blood samples were collected at 0.5, 1, 2, 3, 4, 6, 8, 12, 18, 24, 30, 36, 42, and 48 hours from the tail vein of all the rats after drug treatment. Blood glucose levels were estimated.[8] In the second phase, after 1 day of the respective sulfonylurea treatment, the animals of groups 1 and 4 received esomeprazole 1.8 mg/kg, groups 2 and 5 received esomeprazole 3.6 mg/kg, and groups 3 and 6 received esomeprazole 30 mg/kg for a period of 8 days. During this period the animals had free access to food and water supplied *ad libitum*. On the 7^th^ day, the rats were fasted for 18 hours, with water supplied *ad libitum*. On the 8^th^ day, esomeprazole was administered and the zero-hour blood sample was collected from all the animals of all the groups. The animals of groups 1, 2, and 3 were administered tolbutamide (40 mg/kg) and groups 4, 5, and 6 were given glibenclamide (40 µg/kg) orally. Blood samples were collected at the above-mentioned prefixed time intervals and blood glucose levels were estimated.

### Statistical analysis

The data is presented as mean±standard error of the mean. Analysis was by using Student’s ‘*t*’ test. P≤0.05 is considered as statistically significant.

## RESULTS

**Figure 1:**
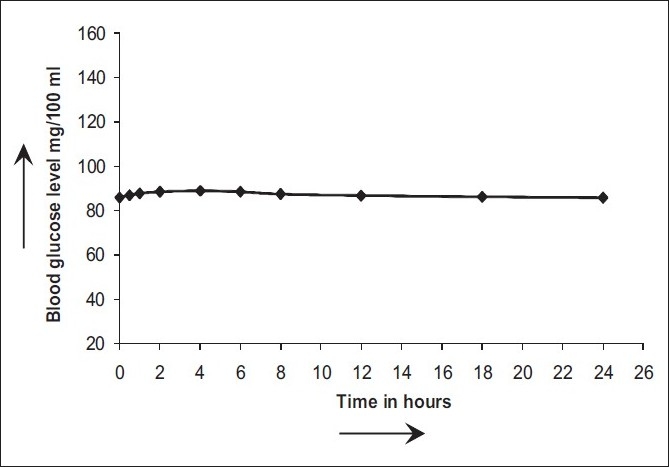
Blood glucose level with esomeprazole (30 mg/kg) in healthy rat

Esomeprazole 30 mg/kg per se did not alter the blood glucose levels [Figure 1]. Tolbutamide 40 mg/kg and glibenclamide 40 µg/kg induced a peak hypoglycemia of 68.47±2.01 mg% and 70.17±2.09 mg%, respectively. The time to onset of hypoglycemia (i.e., the time taken to achieve at least 20% reduction in blood glucose levels) by both the drugs was about 1 hour, and the duration of hypoglycemia (i.e., the duration for which at least 20% reduction in blood glucose level maintained) was 40 h. However, esomeprazole at doses of 1.8 mg/kg and 3.8 mg/kg did not influence the hypoglycemia induced by tolbutamide and glibenclamide. The results are shown in Tables 1 and 2. The pilot studies showed no influence of pretreatment with esomeprazole (up to 20 mg/kg) on the hypoglycemia induced by oral tolbutamide and glibenclamide. Therefore, we tried to assess the influence of more than eight times the therapeutic dose of esomeprazole (i.e., 30 mg/kg) on hypoglycemia induced by sulfonylureas. Tolbutamide- and glibenclamide-induced hypoglycemia was enhanced significantly by pretreatment with esomeprazole at the dose of 30 mg/kg; Pretreatment with esomeprazole enhanced the tolbutamide induced peak hypoglycemia from 59.11±3.78 mg% to 64.53±1.05 mg% and glibenclamide induced peak hypoglycemia from 64.97±1.42 mg% to 72.05±4.93, mg% respectively. Similarly, the duration of hypoglycemia was also enhanced significantly. The results are compiled in.

**Table 1:** Percentage blood glucose reduction with tolbutamide in healthy albino rats before and after esomeprazole

	Esomeprazole (1.8 mg/kg) + tolbutamide (40 mg/kg)		Esomeprazole (3.6 mg/kg) + tolbutamide (40 mg/kg)		Esomeprazole (30 mg/kg) + tolbutamide (40 mg/kg)	
	Before esomeprazole (mean±SEM)	After esomeprazole (mean±SEM)	Before esomeprazole (mean±SEM)	After esomeprazole (mean±SEM)	Before esomeprazole (mean±SEM)	After esomeprazole (mean±SEM)
Fasting	-	-	-	-	-	-
0.5	28.27±3.70	14.16± 2.79***	18.43±4.48	21.34±2.43	8.61±3.08	15.55±2.01*
1.0	43.82±3.90	23.57 ± 2.66***	25.90±4.38	28.58±3.78	24.34±3.78	41.70±2.04***
2.0	50.78±5.43	31.20 ± 2.97***	33.91±2.61	42.00±3.27*	36.51±2.99	36.79±3.71
4.0	59.02±3.53	42.94 ± 1.52***	46.89±3.33	54.29±2.10*	48.27±4.15	52.81±1.45
6.0	68.47±2.01	52.45 ± 2.23***	51.58±1.53	59.12±1.36**	52.47±5.17	56.60±4.54
8.0	56.47 ± 3.44	65.45 ± 1.04**	57.53±3.69	68.02±2.61*	59.11±3.78	61.46±2.36
12.0	39.79 ± 4.62	56.84 ± 3.56**	65.90±1.70	67.22±4.04	47.40±4.05	64.53±1.05**
18.0	33.07 ± 3.93	43.87 ± 3.57*	59.27±4.76	51.17±2.88*	40.18±4.42	53.79±1.33*
24.0	27.30 ± 5.76	33.44 ± 3.92	48.15±5.59	43.92±4.50	36.45±3.21	50.22±2.40**
						
30.0	24.47 ± 5.82	29.76 ± 3.36	37.99±5.99	34.64±3.09	32.17±4.51	49.08±1.11**
36.0	30.59 ± 9.32	22.10 ± 2.99	29.68±4.52	29.37±4.85	26.37±2.19	42.72±4.86**
42.0	20.44 ± 4.47	17.30 ± 1.91	20.96±2.59	16.66±3.77	19.73±1.18	32.39±3.47**
48.0	13.30 ± 3.32	9.45 ± 2.54	9.28±2.22	10.93 ± 2.39	8.18±3.18	20.63±3.53*

* Significant at *P*<0.05;

** Highly significant at *P*<0.01;

*** Very highly significant at *P*<0.001

**Table 2:** Percentage blood glucose reduction with glibenclamide in healthy albino rats before and after esomeprazole

	Esomeprazole (1.8 mg/kg) + glibenclamide (40 µg /kg)		Esomeprazole (3.6 mg/kg) + glibenclamide (40 µg /kg)		Esomeprazole (30 mg/kg) + glibenclamide (40 µg /kg)	
	Before esomeprazole (mean±SEM)	After esomeprazole (mean±SEM)	Before esomeprazole (mean±SEM)	After esomeprazole (mean±SEM)	Before esomeprazole (mean±SEM)	After esomeprazole (mean±SEM)
Fasting	-	-	-	-	-	-
0.5	28.96±3.78	13.58 ± 2.35**	21.75±2.53	17.36± 5.41	16.36±1.72	42.91±1.04***
1.0	44.91±4.02	23.35 ± 2.19***	29.11 ± 3.90	25.71±4.87	38.80±8.81	64.16±0.85**
2.0	52.05±5.59	31.26 ± 2.66**	42.71 ± 3.30	36.16±2.80	42.43±9.72	70.52±0.52**
4.0	60.49±3.65	43.39 ± 1.39***	55.20 ± 1.97	47.56±3.55*	57.51±3.00	–
6.0	70.17±2.09	53.32 ± 2.01***	60.11 ± 1.09	52.69±1.76**	64.97±1.42	72.05±4.93
8.0	58.07±3.55	66.72 ± 1.06*	69.14±2.40	58.49±4.41*	58.66±3.21	65.79±1.84*
12.0	40.77±4.72	57.80 ± 3.57**	68.29 ± 3.80	67.25±2.51	58.38±3.93	56.60±3.86
18.0	33.85±4.02	44.39±3.45*	52.06±2.94	60.60±4.59	50.92±2.79	51.86±1.40
24.0	27.98±5.90	33.61±3.62	44.64±4.59	49.29±5.16	45.68±1.16	–
30.0	25.07±5.95	29.77±3.03	35.18±2.99	38.62±5.40	45.05±2.78	68.68±1.44***
36.0	31.33±9.53	21.73±3.05	29.84±4.92	29.97±3.73	38.35±3.65	58.63±2.91***
42.0	20.93±4.57	16.68±2.58	16.87±3.73	20.70±2.46	37.04±1.28	55.02±1.75***
48.0	13.62±3.39	8.54±3.20	11.06±2.35	8.49±2.19	32.34±2.56	46.74±1.20***

* Significant at *P*<0.05;

** Highly significant at *P*<0.01;

*** Very highly significant at *P*<0.001

## DISCUSSION

Esomoprazole at 30 mg/kg dose did not by itself reduce the blood glucose level, indicating that any interaction with antidiabetic drugs in this study is not of the pharmacodynamic type. In the present study, therapeutic doses of esomeprazole did not influence any of the parameters of the hypoglycemia induced by sulfonylureas. However, at 8 times the therapeutic dose, esomeprazole enhanced the duration of hypoglycemia as well as the peak level of hypoglycemia induced by sulfonylureas. The literature reports reveal that sulfonylureas are metabolized mainly by CYP2C9 and CYP3A.[9] At the dose of 30 mg/kg esomeprazole probably inhibits these isoenzymes. Since it requires eight times the therapeutic doses of esomeprazole to inhibit the CYP isoenzymes that are responsible for metabolism of sulfonylureas, these enzymes apparently have low sensitivity/affinity for esomeprazole.

It may be concluded that during concomitant administration of sulfonylureas and esomeprazole at therapeutic doses, drug–drug interaction does not occur. Therefore, the therapeutic dose and the frequency of administration of sulfonylureas need not be adjusted.
